# Fingolimod increases cellular resistance to HIV-1 infection and limits viral reservoir size in peripheral CD4^+^ T-cells

**DOI:** 10.1371/journal.ppat.1014266

**Published:** 2026-06-03

**Authors:** Elisa Moraga, Núria Climent, Alejandro Sánchez-Molina, Sònia Vicens-Artés, María José Maleno, Gabriel Valero López, Carlos Galera Peñaranda, Juan Ambrosioni, José M. Miró, Josep Mallolas, Helena Albendín-Iglesias, José Alcamí, Sonsoles Sánchez-Palomino

**Affiliations:** 1 AIDS and HIV Infection Group, Fundació de Recerca Clínic Barcelona- Institut d’Investigacions Biomèdiques August Pi i Sunyer (FRCB-IDIBAPS), Barcelona, Spain; 2 Department of Medicine, Universitat de Barcelona (UB), Barcelona, Spain; 3 Centro de Investigación Biomédica en Red sobre Enfermedades Infecciosas CIBER (CIBERINFEC), Instituto de Salud Carlos III (ISCIII), Madrid, Spain; 4 Neurology Department, Hospital Clínico Universitario Virgen de la Arrixaca/IMIB, CSUR Multiple Sclerosis and Clinical Neuroimmunology Unit, Murcia, Spain; 5 Hospital Clínico Universitario Virgen de la Arrixaca/ IMIB, HIV Unit, Murcia, Spain; 6 Infectious Diseases Service, Hospital Clínic de Barcelona, University of Barcelona, Barcelona, Spain; 7 Reial Academia de Medicina de Catalunya (RAMC), Barcelona, Spain; Emory University, UNITED STATES OF AMERICA

## Abstract

**Background:**

Fingolimod, a treatment for multiple sclerosis (MS), decreases autoreactive lymphocytes by lymph node sequestration. *In vitro*, fingolimod decreases HIV-1 infection and viral reservoir (VR) through SAMHD1 phosphorylation inhibition and reducing lymphocyte activation and CD4 expression. We identified an exceptional MS patient infected with HIV-1 (HIV+ MS+) while on fingolimod therapy and we have analyzed its impact on HIV-1 infection *in vivo* and *ex vivo*.

**Methods:**

The case was the HIV+ MS+ patient. Controls were 20 PWH (HIV+ MS-), five HIV-negative donors (HIV-MS) and three HIV-negative MS patients treated with fingolimod (HIV-MS+), as the case reported. VR was quantified by IPDA and HIV-1 intracellular RNAs (icRNAs) by ddPCR in peripheral blood CD4^+^ T-cells. CD4^+^ T-cells were infected *in vitro* with an NL4.3-Renilla strain. Immunophenotype, activation markers and phosphorylated SAMHD1 levels were determined by flow cytometry.

**Findings:**

At diagnosis, HIV+ MS+ viral load was nine-fold lower than HIV+ MS- treated at similar Fiebig stage. One year after ART, HIV+ MS+ showed lower intact and defective VR than the HIV+ MS- control group (28-and six-fold decrease respectively). After three years on ART no intact proviruses were detected in HIV+ MS+ . HIV-1 *in vitro* infection was decreased in HIV+ MS+ and HIV- MS + vs HIV+ MS- and HIV-MS-. CD4^+^ T-cells levels from fingolimod treated patients were lower and showed decreased CD4 expression, lymphocyte activation and SAMHD1 phosphorylation vs HIV- MS-. icRNAs were significantly increased after T-cell activation in the HIV+ MS-, while they were barely detected at resting and activated HIV+ MS+ CD4^+^ T-cells.

**Conclusions:**

We describe a strong restriction to HIV-1 infection and replication *in vivo* and *ex vivo* leading to indetectable intact VR in HIV+ MS+ after three years of ART. Potential mechanisms of restriction are CD4 downregulation, T-cell activation inhibition, and SAMHD1 activity enhancement.

## Author summary

In this study, we investigated how a multiple sclerosis treatment called fingolimod impacts HIV-1 infection by following a unique clinical context. Fingolimod works by trapping immune cells in lymph nodes to prevent them from attacking the central nervous system. While previous work suggested this drug blocks HIV-1 infection *in vitro*, studies with SIV and monkeys showed limited success, creating a “translational gap”. We describe the first case of an individual who acquired HIV-1 while already taking fingolimod for multiple sclerosis. This exceptional scenario allowed us to analyze the drug’s impact in a person for the first time. We found that fingolimod created a powerful barrier against HIV-1: this individual had an unusually low viral load and, after three years of standard treatment, no detectable intact viral reservoir remained. Our work shows that the drug restricts HIV-1 by lowering the levels of the virus’s entry receptor on cells and boosting the body’s natural restriction factors. By proving that these effects occur in a human, we bridge the gap between *in vitro* evidence and clinical practice. These findings provide new insights into HIV-1 biology with a clinical approach in the search for a functional cure in HIV-1 infection.

## Introduction

Multiple sclerosis (MS) is a chronic autoimmune disease characterized by demyelination, inflammation, neuronal loss, and gliosis. In early stages, there is infiltration of immune cells from the periphery into central nervous system (CNS), including T- and B-lymphocytes, macrophages, and natural killer cells (NKs). Th1 and Th17 lymphocytes are the main mediators of pathology in MS leading to inflammation and myelin destruction [1–3]. Anti-inflammatory T-helper lymphocytes (Th2) also play a role in the pathogenesis releasing cytokines that attract macrophages and microglial cells. The autoreactive cytotoxic T-cells stimulate B response and the production of anti-myelin antibodies [4]. This produces CNS damage by the immune system leading to the neurodegenerative process that causes MS. For this reason, MS is treated with immunosuppressive medication as fingolimod aimed at the control of cytotoxic T responses [5].

Fingolimod, clinically approved for treatment of MS, is a sphingosine-1-phosphate receptors (S1PR) ligand, modulating different functions at immunological levels [5]. The natural ligand of S1PR is sphingosine-1-phosphate (S1P), a homeostatic factor in the control of lymphocyte circulating levels. S1P is released by lymphatic endothelial cells while S1PR is expressed on lymphocyte cell surface, acting as a signal that induces their egress from the lymphoid organs. Additionally, fingolimod undergoes an intracellular phosphorylation by cellular sphingosine kinases. Once phosphorylated, fingolimod can join S1PR, driving receptor internalization and degradation. Hence, the lack of S1PR on lymphocytes membranes prevents lymphoid tissues egression [6,7]. Consequently, fingolimod results in lymphopenia with a reduction of trafficking of Th and Treg lymphocytes by kidnapping them on lymphoid organs and not allowing them to reach the CNS. Fingolimod also decreases B-cells in blood and CD4/CD8 ratio because CD4^+^ T-cells reduction in circulation is more severe than for CD8^+^ T-cells. Previous works show that NKs and monocytes are less affected because they do not depend on S1PR to be present in blood and their proportions increase in fingolimod-treated patients [8].

Besides its SIPR1-dependent mechanism of action, fingolimod causes an aberrant activation of NFAT1, AP1 and NFκB and induces epigenetic changes in human T cells resulting in impaired TCR-dependent activation in T lymphocytes [9]. In addition, S1PR1 is highly co-expressed with the HIV-1 co-receptor CCR5 on CD4^+^ T-cells and both molecules are internalized jointly when fingolimod is added *in vitro* [10].

Fingolimod has also been studied in the context of HIV-1 infection. The infection of CD4^+^ T-cells by HIV-1 depends mainly on the state of cellular activation, so immunosuppressive drugs inhibiting lymphocyte activation may have an antiviral role against HIV-1, as previously described for tyrosine kinase inhibitors [11], mycophenolate mofetil [12] or cyclosporine A [13] among others. Previous studies with fingolimod and HIV-1 infection have been restricted to *in vitro* testing or in non-human primate (NHP) models. *In vitro* fingolimod increases resistance to HIV-1 infection by decreasing inactivated phosphorylated SAMHD1 (pSAMHD1), an HIV-1 restriction factor, and by reducing CD4 surface density and CCR5 expression, which inhibits viral binding and fusion. Consequently, there is a decrease in infection and establishment of HIV-1 reservoirs [10,14] In NHP, fingolimod retains cytotoxic cells in the lymph nodes decreasing SIV infection of follicular helper lymphocytes, one major reservoir, but a clear impact on proviral levels has not been demonstrated [15,16].

Latently infected CD4^+^ T-cells are the major viral reservoir, which contains integrated HIV-1 DNA copies that can become reactivated following immune activation and are not accessible to current ART [17–19]. On the other hand, HIV-1 reservoir also could be transcriptionally active despite ART [20]. Accordingly, to achieve new antiviral strategies to tackle the HIV-1 reservoir is one major objective in current HIV-1 research [21].

The “block and lock” strategy is an emerging therapeutic approach in HIV-1 cure research that seeks to permanently and profoundly silence the latent viral reservoir. Unlike other strategies such as “shock and kill”, this approach does not attempt to eliminate the virus, but rather to “block” transcriptionally active reservoir and “lock” it in a state of latency [22]. Fingolimod could be potentially a mixed therapeutic drug, both targeting early ad post integration HIV-1 replication stages. It should not be considered a classical “block and lock” agent as its previously reported mechanism of action is inhibiting viral entrance by co-internalization of S1PR1 with HIV-1 receptors (CD4 and CCR5) and a decrease in pSAMHD1, which consequently reduces proviral DNA integration. Nevertheless, it could act as a “block and lock” agent by causing an impaired TCR activation [9], essential for HIV-1 infection [23] and transcription [24].

This exceptional case study involves a MS patient who became HIV-1 infected during treatment with fingolimod. The main objective of this work is to study the impact of fingolimod treatment on the viral reservoir and HIV-1 infection *in vivo* and *ex vivo* in this MS patient.

## Methods

### Ethics statement

All participants gave informed written consent to participate in this study. The study received approval by Ethics Committee of Hospital Clínico Universitario Virgen de la Arrixaca (6-7-2023-HCUVA).

### Case and controls

The reported case (HIV+ MS+) is a bisexual male who was diagnosed of MS at 21 years old in 2011. Treatment with betaferon was initiated in June 2011 and switched to natalizumab in December 2011. Natalizumab was discontinued in March 2013 due to the detection of positive anti-JCV antibodies. In March 2014, treatment with fingolimod was started, which was the only active medication against MS at the time of the HIV diagnosis. He has not suffered new MS outbreaks since the beginning of fingolimod treatment. HIV-1 last negative serology was from 2017 and at this time, he presented a fingolimod associated lymphopenia (400 cells/mm^3^, S1 Table). No additional serological testing was performed between 2017 and 2022. During this period, the patient remained under neurological follow-up and continued treatment with fingolimod. At HIV-1 diagnosis he was asymptomatic. Infection with subtype CRF_01AE was defined. HIV-1 infection was diagnosed in June 2022 by serology (HIV-1 / HIV-2 p24 antigen and antibody ELISA), followed by a confirmatory Western blot showing reactivity to gp41, p24, and gp160, while the p31 band was absent. This banding pattern is consistent with early HIV-1 infection (Fiebig stage V). A subsequent Western blot performed one year after initiation of ART showed a complete pattern, including the presence of the p31 band. These findings support that the diagnosis was made at an early stage (Fiebig V), rather than during a chronic or advanced stage of infection. He presented anal condylomas at diagnosis, was negative for hepatitis B and C and no other sexual transmitted diseases were reported. Plasma HIV-1 viral load and CD4^+^ T-cell-count at baseline were 4.6 Log_10_ copies/mL and 26 CD4^+^ T-cells/mm^3^, respectively. He rapidly started ART with bictegravir/emtricitabine/tenofovir alafenamide (BIC/FTC/TAF), remaining virologically suppressed (<50 copies/mL) during this time and with 122 CD4^+^ T-cells/mm^3^ at three years. CD4^+^ T-cell historical counts and lymphocyte count prior to HIV-1 acquisition can be found in the S1 Table. No HIV-1 associated events have been diagnosed. As part of this observational study, blood samples were taken at one, two and three years after ART initiation.

As controls we selected three MS HIV-1-negative patients treated with fingolimod (HIV- MS+) and five HIV-negative donors (HIV- MS-) from the Banc de Sang i Teixits Barcelona, Spain. HIV- MS+ presented fingolimod associated lymphopenia at similar levels as the HIV+ MS+ case (S2 Table). All the MS subjects were recruited at the Hospital Clínico Universitario Virgen de la Arrixaca, Murcia, Spain. As HIV-positive controls (HIV+ MS-) we included virally suppressed treated PWH from Hospital Clínic, Barcelona, Spain: eight primary infected treated in Fiebig V-VI with BIC/FTC/TAF for one year, seven primary infected and five chronic PWH.

After HIV-1 diagnosis, the HIV+ MS+ patient initiated ART with BIC/TAF/FTC (one tablet once daily), along with acyclovir (200 mg every 8 hours) and trimethoprim–sulfamethoxazole (160/800 mg, one tablet three times per week). In addition, he received clonazepam (0.5 mg) as needed and desvenlafaxine (100 mg orally once daily), a serotonin–norepinephrine reuptake inhibitor (SNRI). Three years after initiating ART, acyclovir was discontinued and folic acid was added to the regimen. The three HIV- MS+ participants were receiving fingolimod (0.5 mg once daily) without any other relevant treatments.

### Quantification of the HIV-1 reservoirs

Intact Proviral DNA Assay (IPDA) was performed on peripheral blood CD4^+^ T-cells enriched by negative selection (EasySep Human CD4^+^ T-Cell Enrichment Kit, StemCell Technologies) from cryopreserved PBMCs as previously published [25]. CEM_NKRCCR5_ cells were included as negative controls and ACH2 cells carrying one single HIV-1 provirus per cell as positive controls. The limit of detection was of 2.3 copies/10^6^ CD4^+^ T-cells.

### CCR5 genotyping

Genomic DNA of the HIV+ MS+ was extracted from PBMCs using the QIAmp DNA Mini Kit, Qiagen. DNA sample was then digested using XbaI (New England Biolabs). CCR5Δ32 detection reaction was then performed using droplet digital PCR (ddPCR, Bio-Rad QX600) with primers flanking the site of the deletion previously published [26] along with a probe targeting the deletion region of the CCR5 gene (5’TTCCATACAGTCAGTATCAATTCTGGAAGA3´) and a second probe targeting the flanking sequences of the deletion fragment (5’TTCCATACATTAAAGATA3’). A positive control consisting of a CCR5 / CCR5Δ32 sample confirmed by NGS was used.

### In vitro infection assay

Infection was performed in CD4^+^ T-cells obtained by enriched negative selection from cryopreserved PBMCs. CD4^+^ T-cells were seeded at a concentration of 10^6^ cells/mL in R20 culture media (RPMI supplemented with glutamine (Corning Mediatech), 20% fetal bovine serum (Biowest) and antibiotics (penicilin 100 UI/mL and streptomicin 100 μg/mL; Lonza)). Cells were expanded with 100 IU/mL of recombinant interleukin-2 (IL-2) (Chiron Corporation) and with αCD3/CD28 (10 µL/10^6^ cells, Dynabeads Human T-Expander CD3/CD28, Gibco) for 72 hours. Afterwards, cultures (10^5^ cells/well) were infected with the X4-tropic HIV-1 clone NL4.3-Renilla [27] (10^5^ RLUs/well). Renilla reporter activity was quantified in cell lysates after 72 hours of infection using Renilla Luciferase Assay System (Promega) in a Xenius luminometer (Safas).

### PBMCs phenotyping

PBMCs were stained with a combination of anti-human antibodies to evaluate lymphocyte subsets (S3 Table). Cells were acquired on the Aurora spectral cytometer (Cytek) using SpectroFlo software (Cytek). Analysis was conducted with FlowJo software (BD Biosciences).

### Quantification of CCR5 at the cell surface

PBMCs were stained with a combination of anti-human antibodies to evaluate CCR5 expression in CD4^+^ T-cells (S4 Table). Cells were acquired on the CytoFLEX V5-B5-R3 (Beckman Coulter). Analysis was conducted with FlowJo software (BD Biosciences).

### Characterization of CD4 activation and SAMHD1 phosphorylation levels by flow cytometry

The characterization was performed in PBMCs under basal and post-activation conditions. Cells were expanded in culture with 0.5μg/mL phytohemmaglutinin (PHA) (Sigma Aldrich) and 100 IU/mL IL-2 for 72 hours and stained for pSAMHD1 and CD25. Cell viability was determined with LIVE/DEAD Blue Fixable Cell Viability Assay (Thermo Fisher Scientific). PBMCs were stained extracellularly with a combination of anti-human antibodies *(S5 Table)* and fixed with BD Cytofix/Cytoperm Fixation Buffer (BD Biosciences). Cells were then permeabilized with methanol, blocked with 10% AB serum and labelled with pSAMHD1 antibody ((Phospho-SAMHD1-Thr592) PE, Cell Signaling). Acquisition was performed in a BD LSRFortessa Flow Cytometer and analysed by FlowJo software. The gating strategy is shown on S1 Fig.

### Intracellular HIV-1 RNA quantification

Peripheral blood CD4^+^ T-cells were enriched from PBMCs by negative selection and seeded at a concentration of 10^6^ cells/mL in R20 medium. Cells were maintained in a resting state or were activated with 100 UI/mL IL-2 and αCD3/CD28 microbeads (10 µL/10^6^ cells). After 96 hours of culture CD4^+^ T-cells were pelleted and RNA was extracted (RNeasy Mini Kit, Qiagen) from resting and activated CD4^+^ T-cells. 5′elongated (LongLTR), unspliced (Pol), polyadenylated (PolyA), and multiply-spliced (TatRev) HIV-1 intracellular RNA (icRNA) were quantified with the One-Step RT-ddPCR Advanced Kit for Probes (Bio-Rad) [20]. Every well contained a control of cellular mRNA presence for CD3 gene as housekeeping [28]. RNA input for all the assays was between 100–600 ng per well (QX600, Bio-Rad). Analysis was performed by adjusting ddPCR RNA raw data concentrations with RNA quantity and concentration in each well. HIV-1 RNA copies were normalized to copies of 10^6^ CD4^+^ T-cells by calculating RNA ng/cell. The limit of detection was of 10.6, 5.8, 3.2 and 4.5 copies/10^6^ CD4^+^ T-cells for LongLTR, Pol, PolyA and TatRev assays respectively.

### Viral isolation assay

Peripheral blood CD4^+^ T-cells were enriched from PBMCs by negative selection CD4^+^ T-cells and were half pelleted (resting) and half seeded at a concentration of 106 cells/mL in R20 medium. Cells were activated in culture with 100 UI/mL IL-2 and αCD3/CD28 microbeads (10 µL/106 cells). After 96h of culture CD4^+^ T-cells were pelleted. CD4^+^ T-cells pellets were stored at -80ºC. Culture supernatants after 96 hours were also stored at -80ºC. One well from the HIV+ MS+ was maintained and co-cultured with CEM_NKRCCR5_ cells in fresh R20 media containing 100 IU/mL IL-2. After 7-, 14- and 21-days supernatants were collected and HIV-1 RNA load was quantified by reverse transcription-quantitative PCR (RT-qPCR) (COBAS Taq-Man HIV-1 test, Roche Molecular Systems). The detection limit for this assay was 50 copies/mL.

### Statistical analysis

Mann-Whitney test was performed to compare data sets with N > 1. We performed a one sample Wilcoxon test to compare sets of data with N > 1 from control groups with the HIV+ MS+ unique data.

P-values are shown in the figures and text. Analysis and graphical representations for figures was performed with GraphPad Prism 10 software.

## Results

### Evolution of viral load and reservoirs in HIV+ MS+ treated with fingolimod

**Fig 1:**
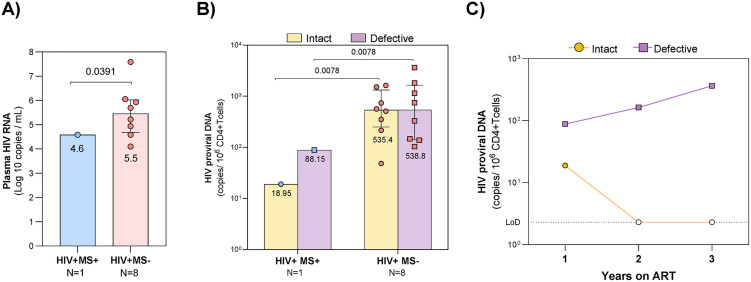
Plasma viral load and HIV-1 reservoir in fingolimod-treated HIV-1 patient and PWH controls. **A.** Viral load (copies/mL) was quantified at diagnosis by RT-qPCR in HIV+ MS+ case (blue circles) and eight HIV+ MS− individuals at Fiebig stages V–VI (red circles). Median values and interquartile ranges are shown. **B.** HIV-1 reservoir in peripheral blood CD4 ⁺ T-cells after 48 weeks on ART. Intact and defective proviral DNA (copies/10⁶ CD4 ⁺ T-cells) were quantified by IPDA in HIV+ MS+ (blue circles) and in eight HIV + MS− individuals at Fiebig stages V–VI (red circles). Median values and interquartile range are shown. **C.** HIV-1 reservoir in the HIV+ MS+ peripheral blood CD4 ⁺ T-cells during the three years of follow-up after ART initiation. Intact and defective proviral DNA (copies/10⁶ CD4 ⁺ T-cells) were quantified by IPDA, open symbols are values under the limit of detection (LoD). One sample Wilcoxon test was performed to compare the HIV+ MS+ values vs HIV+ MS- group.

Viral load of the HIV+ MS+ at the time of HIV-1 diagnosis was 4.6 Log_10_ copies/mL (Fig 1A) almost 10 times significantly lower (p = 0.0391) than the median VL of eight HIV+ MS- PWH diagnosed at the same Fiebig V-VI stage (median (IQR) basal viral load of 5.5 (4.6-6.02) Log_10_ copies/mL). At baseline the HIV+ MS+ displayed low CD4 levels (26 CD4^+^ T-cells/mm^3^) because of fingolimod treatment. The control HIV+ MS- group at similar Fiebig stage had a median (IQR) of 395 (343-600.3) CD4^+^ T-cells/mm^3^. After one year on ART, the HIV+ MS+ patient was virologically suppressed on ART and CD4 count increased two-fold (53 CD4^+^ T-cells/mm^3^). Virological suppression was maintained after two and three years on ART and CD4^+^ T-cell counts were 50 and 122 cells/mm^3^, respectively.

To assess fingolimod impact on HIV-1 reservoir we quantified proviral DNA by IPDA in peripheral blood CD4^+^ T-cells from the HIV+ MS+ case and eight HIV+ MS- controls (Fiebig V-VI) after one year on ART with BIC/FTC/TAF. The HIV+ MS+ presented 18% of intact reservoir (18.95 copies/10^6^ CD4^+^ T-cells), while 82% of the reservoir was defective (88.15 copies/10^6^ CD4^+^ T-cells). In contrast, the HIV+ MS- group showed a 28-fold significantly higher intact reservoir (p = 0.0078) as compared to the HIV+ MS+ patient with median (IQR) of 535.4 (249.4-1321) copies/10^6^ CD4^+^ T-cells. Similarly, HIV+ MS- group had a median (IQR) of 538.8 (140.1-1650) defective copies/10^6^ CD4^+^ T-cells, which represents a six-fold significant increase (p = 0.0078) compared with defective copies in the HIV+ MS+ patient (Fig 1B)*.* The intact reservoir in the HIV+ MS+ case decreased during follow-up and after two and three years of ART no intact copies were detected (Fig 1C). There were 162.89 and 363.62 defective copies/10^6^ CD4^+^ T-cells after two and three years on ART respectively.

### CCR5 genotype from the HIV + MS+ participant

We characterized CCR5 genotype by ddPCR to exclude the possibility that the patient’s clinical course was influenced by the Δ32 mutation. The genotype confirmed that the HIV+ MS+ participant is homozygous for the wild-type allele.

### Fingolimod restricts HIV-1 infection in vitro

**Fig 2:**
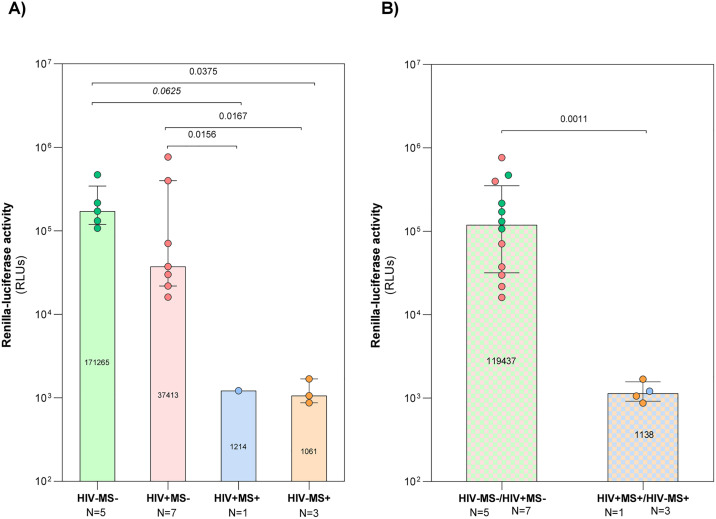
*In vitro* HIV-1 infectivity assays. **A.** Replication of HIV-1 NL4.3 *Renilla* in peripheral blood CD4 ⁺ T-cells activated for 72 hours in the four groups: non-infected controls (HIV− MS − , green circles), PWH (HIV+ MS − , red circles), case report (HIV+ MS + , blue circles), and MS patients treated with fingolimod (HIV− MS + , orange circles). **B.** Comparison between fingolimod non treated (HIV- MS-/HIV+ MS-) and treated (HIV+ MS + /HIV- MS+) participants. Viral replication was quantified by relative luminescent units (RLUs). The graph shows median values and interquartile ranges. Mann-Whitney test was performed to compare the different groups with N > 1. One sample Wilcoxon test test was performed to compare the HIV+ MS+ values vs HIV+ MS- group.

To assess the potential cellular restriction elicited by fingolimod, we evaluated *in vitro* infection with an infectious CXCR4 tropic HIV-1 clone NL4.3 carrying the renilla-luciferase gene as reporter. The infection assay was performed in purified and activated peripheral blood CD4^+^ T-cells from five non-infected controls (HIV- MS-), the HIV+ MS+ case after one year on ART, seven PWH virally suppressed (HIV+ MS-) and three patients with multiple sclerosis and no HIV infection treated with fingolimod (HIV- MS+). The RLUs data were obtained from infection assays performed directly on previously enriched and activated CD4^+^ T-cells rather than PBMCs. By using a fixed and standardized number of purified target cells per well across all conditions, the need for further normalization based on CD4 frequency among PBMCs (which may be very different between participants because of fingolimod) is bypassed. To ensure data accuracy, the following steps were taken. The blank measurement (background signal) was subtracted from all raw RLUs values. RLUs medians (IQR) from HIV- MS- participants 171265 (119437–343141) were significantly higher than HIV- MS + 1061 (874–1685) (p = 0.0375) and did not reach significancy but tendency to be lower than the HIV+ MS+ (1214; p = 0.0625). RLUs medians from HIV+ MS- participants 37413 (21824–397543) were significantly higher than HIV-MS + 1061 (874–1685) (p = 0.0167) and the HIV+ MS+ (1214; p = 0.0156). There were no significant differences between HIV- MS- and HIV+ MS-, nor between HIV + MS+ and HIV-MS- (Fig 2A). If we group participants treated with fingolimod (HIV- MS- and HIV+ MS-) and not treated with fingolimod (HIV+ MS+ and HIV- MS+), the medians were significantly different (p = 0.0011) with 1138 (921–1567) and 119437 (31808–352243) RLUs respectively (Fig 2B). We determined the median RLU value of the participants not taking fingolimod and set it as 100%. We then calculated the percentage represented by the median of the fingolimod-treated group relative to this baseline. This, represents a 0.9% of Renilla-luciferase activity in fingolimod-treated group in comparison with non-treated.

### PBMC subsets in fingolimod-treated-patients

**Fig 3:**
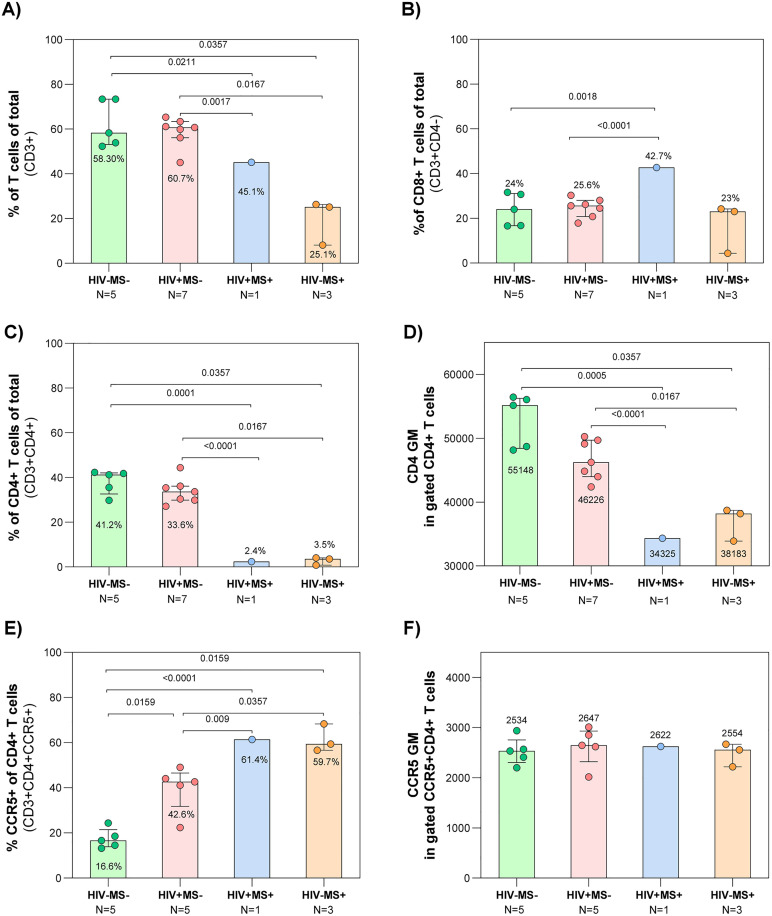
Phenotypic characterization of T cell subsets. Flow cytometry analysis of PBMCs from non-infected controls (HIV− MS− , green circles), PWH (HIV+ MS− , red circles), case report (HIV+ MS+ , blue circles), and MS patients treated with fingolimod (HIV− MS+ , orange circles). The graph shows median values and interquartile ranges. Mann-Whitney test was performed to compare the different groups with N > 1. One sample Wilcoxon test was performed to compare the HIV+ MS+ values vs HIV+ MS- group. **A.** Percentage of CD3^+^T-cells. **B.** Percentage of CD8 ⁺ T-cells. **C.** Percentage of CD4 ⁺ T-cells. Proportions in Panels A, B, and C were calculated using total alive cells as the denominator. **D.** Geometric mean fluorescence intensity (GM) of CD4 expression in gated CD4 ⁺ T-cell population. **E.** Percentage of CCR5^+^ cells among CD4 ⁺ T-cells. **F.** Geometric mean fluorescence intensity (GM) of CCR5 expression in gated CCR5^+^ CD4 ⁺ T-cell population.

We obtained significantly lower percentages of T-cells in the HIV+ MS+ participant (45.1%) versus the HIV-MS- (58.3%) (p = 0.0211) and the HIV + MS- (60.7%) (p = 0.0017). Similarly, the HIV- MS+ group presented significantly lower T-cells (25.1%) compared with HIV- MS- (p = 0.0357) and HIV+ MS- (p = 0.0167) (Fig 3A). CD8^+^ T-cells only showed significant differences between the HIV+ MS+ participant (42.7%) and the HIV-MS- (24%) (p = 0.0018) and the HIV+ MS- (25.6%) (p < 0.0001) (Fig 3B). CD4^+^ T-cells were reduced in patients treated with fingolimod (2.4% in the HIV+ MS+ and 3.5% in the HIV- MS+). The HIV+ MS+ participant was significantly decreased from HIV- MS- (41.2%) (p = 0.0001) and from HIV+ MS- (33.6%) (p < 0.0001). Similarly, the HIV- MS+ group presented significantly lower CD4^+^ T-cells compared with HIV- MS- (p < 0.0357) and HIV+ MS- (p = 0.0167) (Fig 3C). Besides, expression of CD4 on the surface of CD4^+^ T-cells as determined by geometric mean (GM) of fluorescence was significantly lower in CD4^+^ lymphocytes from the HIV+ MS+ patient (GM = 34325) versus the HIV- MS- (55148) (p = 0.0005) and the HIV+ MS- (46226) (p < 0.0001). Similarly, the HIV- MS+ group presented significantly lower GM (38183) compared with HIV- MS- (p = 0.0357) and HIV+ MS- (p = 0.0167) (Fig 3D).

To assess if fingolimod was also decreasing the expression of the CCR5 HIV-1 coreceptor, we compared the percentages of CCR5^+^ and GM fluorescence intensity on CD4^+^ T-cells. Significant differences were observed in CCR5 expression across the study groups. The control group (HIV- MS-) displayed the lowest frequency (16.6%), which significantly increased to 42.6% in HIV+ MS- participants (p = 0.0159), to 61.4% in the HIV+ MS+ case (<0.0001) and to 59.7% in the HIV- MS+ participants (p = 0.0159). Notably, fingolimod-treated patients, both the HIV+ MS+ case and the HIV- MS+ group displayed significantly higher CCR5 levels compared to the HIV+ MS- group (p = 0.009 and p = 0.0357, respectively) (Fig 3E). The membrane CCR5 expression levels assessed by GM presented no significant differences among the four groups (Fig 3F).

**Fig 4:**
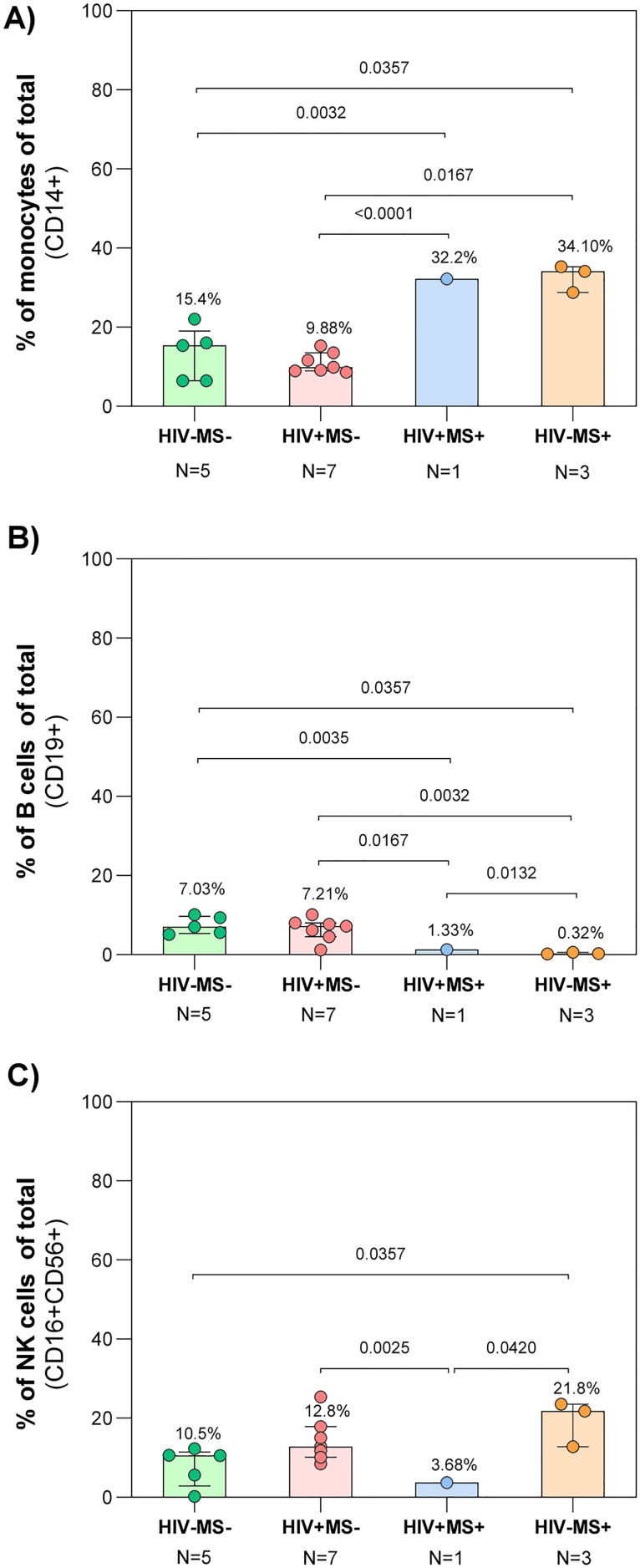
Phenotypic characterization of PBMC subpopulations. Flow cytometry analysis of PBMCs from non-infected controls (HIV− MS− , green circles), PWH (HIV+ MS− , red circles), case report (HIV+ MS+ , blue circles), and MS patients treated with fingolimod (HIV− MS+ , orange circles). The graph shows median values and interquartile ranges. Mann-Whitney test was performed to compare the different groups with N > 1. One sample Wilcoxon test test was performed to compare the HIV+ MS+ values vs HIV+ MS- group. **A.** Percentage of monocytes defined as CD14^+^. **B.** Percentage of B-cells, defined as CD19^+^. **C.** Percentage of NK cells, defined as CD16^+^CD56^+^. Proportions in Panels A, B, and C were calculated using total alive cells as the denominator.

Monocytes were significantly higher in the HIV+ MS+ participant (32.2%) compared with HIV- MS- (15.4%) (p = 0.0032) and from HIV+ MS- (9.88%) (p < 0.0001). Similarly, the HIV- MS+ group presented significantly higher monocyte percentage compared with HIV- MS- (p = 0.0357) and HIV+ MS- (p = 0.0167) (Fig 4A). B lymphocytes were significantly decreased in participants from the HIV-MS+ group (0.32%) compared with HIV+ MS+ (1.33%) (p = 0.0132), HIV- MS- (7.03%) (p = 0.0357) and HIV+ MS- (7.21%) (p = 0.0032). The HIV+ MS+ participant had lower B cells levels than the HIV- MS- (p = 0.0035) and HIV+ MS- (p = 0.0167) (Fig 4B). NKs percentage in the HIV+ MS+ case (3.68%) was significantly decreased versus HIV- MS+ (21.8%) (p = 0.042) and HIV+ MS- (12.8%) (p = 0.0025) (Fig 4C).

We quantified the GM fluorescence intensity (GM) of additional surface markers (CD3, CD19 and CD57) (S2 Fig). CD3 GM was significantly reduced in CD3^+^ T-cells from HIV+ MS+ individuals compared with HIV+ MS+ controls (p = 0.0162) and showed a slight decreasing trend in HIV+ MS+ patients versus HIV+ MS- (p = 0.07). Moreover, HIV+ MS+ individuals also exhibited a significantly lower CD3 expression level compared with HIV- MS+ controls. CD19 GM in B cells was significantly lower in fingolimod-treated individuals and in HIV+ MS- group relative to HIV- MS- controls. CD57 GM was increased in CD4^+^ T-cells in the fingolimod-treated individuals in comparison with HIV- MS- participants. To analyze if the increase in the CD57 GM (a senescence biomarker) was due to an increase in the percentage of CD57^+^CD4^+^ T-cells, we analyze this subset. The results show that, in fact, there is an increase in the CD57^+^CD4^+^ T-cells subset in the fingolimod-treated individuals in comparison with both HIV- MS- and HIV+ MS- group. Together, these data reveal selective alterations in CD3, CD19 and CD57 surface expression patterns in circulating lymphocytes of HIV+ MS+ and HIV- MS+ individuals receiving fingolimod.

### Fingolimod interferes with lymphocyte activation and SAMHD1 phosphorylation

**Fig 5:**
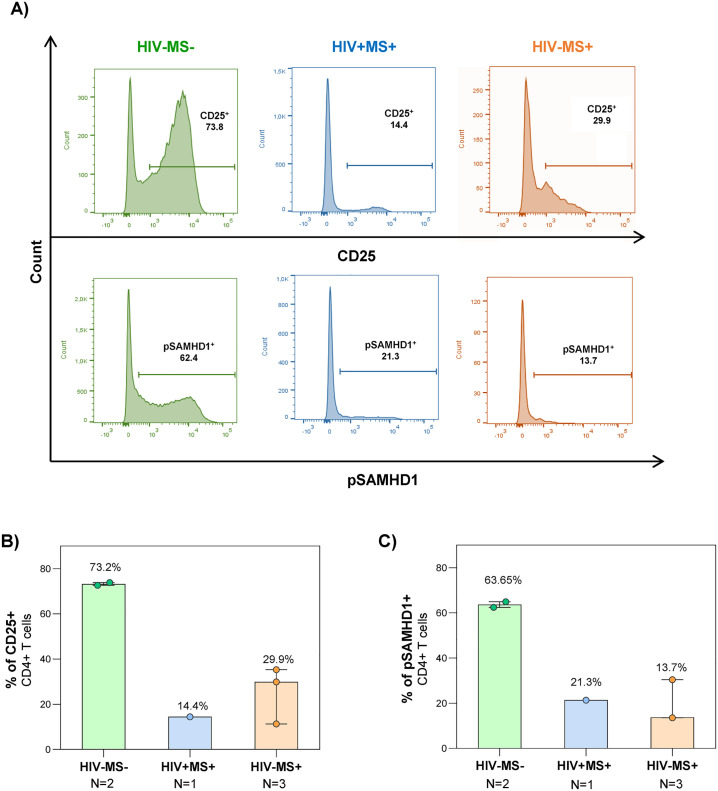
Expression of CD25 and pSAMHD1 in activated PBMCs. Flow cytometry analysis of PBMCs activated for 72h with PHA + IL-2, from non-infected controls (HIV− MS− , green), case report (HIV+ MS+ , blue), and MS patients treated with fingolimod (HIV− MS+ , orange). **A.** Representative flow cytometry histograms for CD25 and pSAMHD1 determination in previously gated CD4^+^ T-cells (see S1 Fig) for each experimental group. Particpants with values near to medians were selected for the plots. **B**. Percentage of activated CD4 ⁺ T-cells determined by CD25 surface expression. **C.** Percentage of pSAMHD1 in CD4 ⁺ T-cells. The graph shows median values and interquartile ranges.

To assess the impact of fingolimod on CD4^+^ T-cell activation and on SAMHD1 restriction activity we analyzed CD25 expression and SAMHD1 phosphorylation in CD4^+^ T-cells after 72h of stimulation with PHA and IL-2 by flow cytometry (Fig 5A). The levels of activated CD4^+^ T-cells, were higher in HIV- MS- controls (73.2%) than in patients treated with fingolimod (14.4% in HIV+ MS+ and 29.9% in HIV- MS+) (Fig 5B*)*. Similarly, pSAMHD1 levels in activated CD4^+^ T-cells from patients under fingolimod were decreased (21.3% in the HIV+ MS+ and 13.7% in the HIV- MS+) in comparison with HIV- MS- (63.65%) (Fig 5C).

### Fingolimod blocks RNA expression and HIV-1 reactivation in peripheral blood CD4 lymphocytes

**Fig 6:**
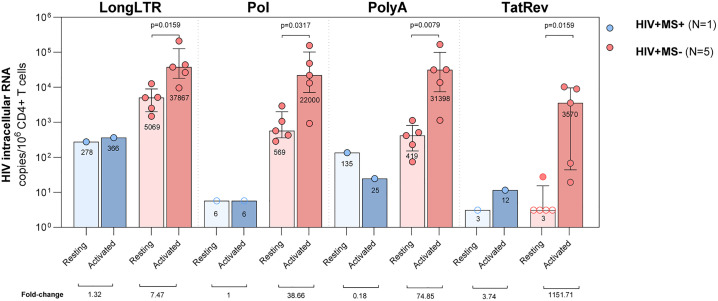
Transcriptionally active reservoir on resting and activated peripheral blood CD4^+^ T-cells. Intracellular RNA was quantified by ddPCR in the HIV+ MS+ (case report, blue circles) and five PWH on ART with undetectable viral load (HIV+ MS-, red circles). The graph shows median values and interquartile ranges of different RNAs: 5’elongated (LongLTR), unspliced (Pol), polyadenylated (PolyA) and multiply spliced (TatRev) HIV-1 intracellular RNA in copies/10⁶ CD4^+^ T-cells. Quantification was performed in resting and peripheral blood CD4^+^ T-cells activated with αCD3/CD28 + IL-2 for 96 hours. Statistical Mann-Whitney test was performed between resting and activated conditions in HIV+ MS-. Significant p-values are shown. Fold changes between both conditions are shown under the graph. Open symbols represent samples that were under the limit of detection.

HIV-1 icRNA in resting and CD4^+^ T-cells activated with αCD3/CD28 and IL-2 was analyzed by ddPCR in the HIV+ MS+ individual and five virologically suppressed PWH on ART (Fig 6). As for proviral reservoir, transcriptionally active and inducible reservoir was only characterized in peripheral blood. In the PWH group, after activation there was a significant increase for all HIV-1 RNAs analyzed: Long LTR (eight-fold from 5069 to 37867 copies/10^6^ CD4^+^ T-cells; p = 0.0159), Pol (38-fold from 569 to 22000 copies/10^6^ CD4^+^ T-cells; p = 0.0317), PolyA (75-fold from 419 to 31398 copies/10^6^ CD4^+^ T-cells; p = 0.0079) and TatRev (1151-fold from 3 to 3570 copies/10^6^ CD4^+^ T-cells; p = 0.0159) In contrast, basal HIV-1 icRNAs were much lower in the HIV+ MS+ individual and there was not or bare increase after cellular activation: LongLTR (from 278.1 to 366.1 copies/10^6^ CD4^+^ T-cells), Pol (no increase), PolyA (decrease from 135 to 25 copies/10^6^ CD4^+^ T-cells) and TatRev (from 3 to 12 copies/10^6^ CD4^+^ T-cells).

We also analyzed the transcription levels normalized to DNA to better estimate the transcriptional output in the different individuals. Our analysis reveals two key findings: firstly, basal resting transcription levels are consistently low across majority of the participants, with the RNA/DNA ratio remaining below 1. Upon activation, while the majority of control participants showed a marked increase in transcriptional output, the fingolimod treated participant exhibited a clear transcriptional block, failing to increase the RNA/DNA ratio significantly (S3 Fig).

In parallel, we measured HIV-1 RNA levels in culture supernatant after 96 hours of activation with αCD3/CD28 and IL-2, (S4 Fig)*.* There was a strong correlation between HIV-1 viral load in culture supernatants and icRNA levels in the PWH group whereas no detection was observed in the HIV+ MS+ patient. Accordingly, we could not isolate the virus from the fingolimod-treated patient after co-culture with CEM_NKRCCR5_ for 21 days, suggesting no virus production after peripheral blood CD4^+^ T-cell activation.

## Discussion

Herein, we have presented a HIV+ MS+ case who was treated with fingolimod and became infected by HIV-1 thereafter. Interestingly, in comparison with PWH treated at similar Fiebig stages he presented a ten-fold reduction in viral load before ART and a 28-fold reduction in intact viral reservoir one year after ART. The proportion of intact proviruses in the peripheral compartment after one year of ART was surprisingly low (18%) in the HIV+ MS+ compared with the HIV+ MS- (50%). In patient´s evolution intact proviral DNA was not detected after two and three years on ART although a large number of CD4^+^ T-cells should be analyzed to conclude significant decrease in the intact reservoir. In addition, we could not reactivate viral transcription and isolate the virus in culture. Two non-exclusive possibilities could explain these findings: either the cells are refractory to viral reactivation and infection, which is directly linked to lymphocyte activation and SAMHD1 restriction, or the reservoir size outside the lymphatic compartments has decreased below a critical threshold, preventing the detection of intact virus or its reactivation in peripheral blood.

Regarding the possibility of a Δ32 mutation cold be influencing the surprisingly low reservoir and viral loads at primoinfection, we performed CCR5 genotyping [26]. We confirmed, the absence of the mutation and consequently its lack of impact on the HIV+ MS+ clinical course. We also quantified CCR5 surface levels on patient’s lymphocytes and compared it with the three control groups used along the whole work. Data showed significant higher levels of CCR5 percentages in circulating CD4^+^ T-cells in participants with MS who were taking fingolimod. Other studies previously reported that CCR5^+^ T-cells are increased in MS, which matches our results [29]. Furthermore, CD4^+^ T-cells upregulate CCR5 upon lymphocyte differentiation, which correlates with their acquisition of an effector memory phenotype. Consequently, CCR5 expression was previously associated with T_EM_ and T_EMRA_ CD4^+^ T-cell subsets [30]. Actually, it has been described that fingolimod increases proportion of T_EM_ and T_EMRA_ CD4^+^ T-cell subsets in peripheral blood because of the sequestration mainly of CCR7 ⁺ T_CM_ and T naïve CD4^+^ T-cells inside lymph nodes [31,32]. Additionally, the increase found under fingolimod in the GM and percentage of CD57 in blood peripheral CD4 ⁺ T-cells reflects expansion of T_EM_ and T_EMRA_ subsets, which also express CD57 senescent marker [33]. This, together with our findings suggests that CD4^+^ T-cells subsets in peripheral blood may be altered by fingolimod, by increasing the proportions of T_EM_ and T_EMRA_ CD4^+^ T subsets indicating that an altered circulating T‑cell composition could also coexist with a direct drug effect in other receptors on CD4^+^ T-cells [10]

Second, fingolimod interferes with TCR-dependent activation [9] of CD4^+^ T-cells which is essential for viral infection [23] and reactivation from latency [24]. Third, decreased SAMHD1 phosphorylation in activated CD4^+^ T-cells in the presence of fingolimod increases cellular restriction to HIV-1 infection. It is important to note that these experiments were performed in isolated CD4^+^ T-cells to avoid the bias due to low CD4 counts in PBMCs by CD4 sequestration in lymph nodes. Both a low CD4 expression in the membrane and an ineffective activation of CD4^+^ T-cells are compelling with previously published results [9]. Besides, *in vitro* infection results support a strong resistance to infection is established in CD4^+^ T-cells from patients treated with fingolimod, pointing to the induction of specific restriction factors blocking HIV-1 infection. Of note, infection was performed with an X4-tropic infectious HIV-1 clone to avoid the bias of different CCR5 expression levels in the cell surface [10].

Regarding its impact on immune cells, fingolimod is known to decrease blood B-cell counts and reduce CD4^+^ T-cells more significantly than CD8^+^ T-cells. On the other hand, NK cells and monocytes do not rely on S1PR expression to remain in the bloodstream, and in fact their levels are higher in patients treated with fingolimod. Accordingly, in our study there was a bright decrease in CD4^+^ T-cell and B cells in fingolimod-treated patients. CD8^+^ lymphocytes remained relatively stable across groups, but were higher in the HIV+ MS+ reported case.

The mild reduction in CD3 GM in circulating CD3^+^ T-cells under fingolimod could be explained by partial CD3/CD4 co-internalization or membrane redistribution driven by S1P1, consistent with CD4 surface downregulation [10]. CD4 colocalizes with the TCR/CD3 complex in activated T cells [34], and fingolimod may slightly internalize with both CD4 and TCR/CD3. In addition, enrichment of T_EM_ and T_EMRA_ CD4^+^ subsets, which naturally express lower CD3, may further reduce GM in fingolimod treated people [32,35]. A plausible explanation for the reduction in CD19 GM observed in B cells from individuals treated with fingolimod is due to CD19 translocation to lipid rafts [36]upon activation [37]and partial co-internalization with S1PR. The reduced CD19 GM observed in fingolimod treated patients can also be explained by shifts in circulating B cell subset composition toward low CD19 transitional and exhausted populations, a phenomenon well described in fingolimod treated individuals [38] and consistent with the broader remodeling of B cell subsets under S1P1 modulation [31]. The reduced CD19 GM in HIV-infected individuals likely results from direct gp120-mediated disruption of B-cell raft-associated receptors, reducing CD19 in the B cell membrane [39] and from chronic activation driven expansion of immature/exhausted B-cell subsets with intrinsically lower CD19 density [40]. Our findings therefore extend this concept by suggesting that CD3 and CD19 surface intensity may also be partially reduced in vivo, likely reflecting a combination of altered lymphocyte subset composition, S1P-dependent membrane remodeling, and changes in raft associated receptor distribution. Further mechanistic studies will be required to clarify the basis of this decrease in CD3 and CD19 GM in fingolimod treated patients.

One major limitation of our study is that we described one unique patient but we compared this exceptional case with adequate controls including fingolimod-treated patients. Due to fingolimod-induced sequestration in lymph nodes we may be underestimating the reservoir by only quantifying provirus in peripheral blood. Sequestration of lymphocytes in lymphoid tissues due to fingolimod treatment is a critical factor that limits our ability to extrapolate peripheral blood findings to the whole-body reservoir. Another limitation of this study is that we could not obtain a blood sample before starting ART and therefore we cannot compare the basal reservoir with the control group of PWH infected at the same stage. However, the viral load at diagnosis was one Log below the median viral load detected in HIV-infected patients treated at the same Fiebig stage and the reservoir one year after ART was ten times lower (107 DNA copies/10^6^ CD4^+^ T-cells) as compared to HIV+ MS- patients (1074 DNA copies/10^6^ CD4^+^ T-cells) suggesting that HIV-1 infection was produced at low level. Furthermore, the low proportion of intact proviruses (18%) reflects also this phenomenon. In spite of exclusively having the opportunity to study reservoir in peripheral CD4^+^ T-cells, rather than the total body CD4^+^ T-cells; this work is the first to complete *in vitro* studies and studies performed on NHP about HIV-1 and fingolimod implications in viral reservoir and its dynamics in a human case [10,14–16].

The striking findings reported in the HIV+ MS+ participant should be interpreted in the context of prior NHP studies. In SIV-infected NHP, fingolimod administered transiently at ART initiation induced lymphocyte sequestration and increased cytolytic cell frequencies in lymph nodes but did not reduce intact proviral genomes during ART nor substantially alter plasma viral decay, with only a modest and inconsistent delay in viral rebound after treatment interruption [15,16]. These divergent outcomes likely reflect fundamental differences in fingolimod timing administration. In the human case, fingolimod treatment was already present at the time of HIV-1 infection, which could limit the establishment of viral reservoir, whereas in the macaque study fingolimod was administered after systemic infection. Additionally, Vpx protein in SIV is a critical factor because it is known that it induces the proteasomal degradation of SAMHD1. Since HIV-1 lacks Vpx, SAMHD1 remains protected from degradation, allowing fingolimod to exert its full restrictive effect on viral replication [41–44]. Together, the data suggest that fingolimod’s potential relevance to HIV-1 cure strategies may lie primarily in restricting early infection or reservoir establishment by changing the CD4^+^ T-cell compartment prior to HIV-1 exposure. This limits fingolimod applicability as a therapeutic strategy in individuals already living with HIV-1 with or without ART.

The coexistence of HIV-1 infection and MS appears to be rare, and the available literature is largely limited to isolated case reports and small case series [45–49]. To our knowledge, no cohort studies have specifically evaluated HIV-1 incidence or prevalence in MS patients treated with fingolimod. The only directly relevant data consist of small serological studies in MS cohorts that did not detect HIV-positive cases but were clearly underpowered for epidemiological inference [50–52]. Therefore, our findings should be interpreted as mechanistic and hypothesis-generating, and cannot be directly extrapolated to population-level effects in fingolimod-treated MS cohorts.

We hypothesized, according to previous *in vitro* results [10,14], that this patient had a low probability of infection due to treatment with fingolimod, but despite the inhibitory potential of this drug and its ability to enhance restriction to the virus, he contracted HIV-1 infection. This reflects that fingolimod restriction is not absolute and depending on the balance between protective and increased exposures, chances of infection remain. Besides, we only had the opportunity to quantify the reservoir and characterize all the aspects that restrict infection in peripheral blood cells, but we do not know if these protective mechanisms, CD4 down-regulation, low activation and increased SAMHD1 restriction are active in other tissues such as the GALT system that are preferential anatomical sites of HIV-1 infection. The work carried out with NHP showed these aspects in lymph nodes but did not study other lymphoid organs such as the GALT. It would be interesting as a future perspective to study the GALT reservoir and mechanisms of restriction to HIV-1 infection in this compartment.

A key limitation of this study is the exclusive analysis of peripheral blood CD4 ^+^ T-cells, particularly in the context of fingolimod-induced lymphocyte sequestration. As a result, our findings should be interpreted as reflecting compartment-specific effects on circulating cells, including reduced susceptibility and transcriptional activity, rather than evidence of a reduced total-body HIV reservoir. Nevertheless, the observed phenotype in peripheral blood raises the possibility that similar mechanisms could operate in tissue compartments. This hypothesis remains to be formally tested in studies directly assessing the reservoir across anatomical sites, and will be essential to determine whether such effects could have implications for the use of fingolimod in reservoir-targeting strategies or analytical treatment interruption in the context of functional cure approaches.

In summary, we report the first case of an MS patient who acquired HIV-1 during fingolimod treatment. Our results show that fingolimod restricts HIV-1 infection *in vivo* and *ex vivo* leading to decreased susceptibility to infection and low viral reservoir through different mechanisms as downregulation of CD4 expression, block of CD4^+^ T-cell activation and increased SAMHD1 activity. Fingolimod should not be considered a classical “block and lock” agent as its mechanism of action is inhibiting viral entrance and SAMHD1 phosphorylation, which consequently reduces proviral DNA integration. Nevertheless, in our results we were not able to induce HIV-1 transcription after *ex vivo* activation, that is one milestone of “block and lock” strategy. Therefore, we consider that fingolimod id not a classical “block and lock” agent but a multistage inhibitor acting on early and post integration steps of the viral cycle.

## Supporting information

S1 Table HIV+ MS+ leucocytes, lymphocytes, CD8^+^ T-cell and CD4^+^ T-cell historical counts and HIV-1 status. (DOCX)

S2 Table Annual leucocyte and lymphocyte counts from fingolimod treated participants (HIV+ MS + , HIV- MS+). The values shown are the means at each year.
(DOCX)

S3 Table List of fluorescent antibodies and markers used for phenotyping cytometry analyses. (DOCX)

S4 Table List of fluorescent antibodies and markers used for CCR5 cytometry analyses. (DOCX)

S5 Table List of fluorescent antibodies and markers used for pSAMHD1 and activation cytometry analyses. (DOCX)

S1 Fig Gating strategy for CD4^+^ T-cell activation and pSAMHD1 levels determination. Flow cytometry analysis of PBMCs activated for 72h with PHA + IL-2. It is a representative participant from the HIV- MS- group.
(TIFF)

S2 Fig Surface expression levels of different lymphocyte markers. Flow cytometry analysis of PBMCs from non-infected controls (HIV− MS− , green circles), PWH (HIV+ MS − , red circles), case report (HIV+ MS+ , blue circles), and MS patients treated with fingolimod (HIV− MS+ , orange circles). The graph shows median values and interquartile ranges. Geometric mean fluorescence intensity (GM) was used to measure expression levels on membrane. Mann-Whitney test was performed to compare the different groups with N > 1. One sample Wilcoxon test test was performed to compare the HIV+ MS+ values vs HIV+ MS- group. A. CD3 GM in gated CD3^+^ cells. B. CD19 GM in gated CD19^+^ cells. C. CD57 GM in gated CD3^+^CD4^+^ cells. D. Percentage of CD57^+^ in gated CD3^+^CD4^+^ cells.
(TIFF)

S3 Fig Transcriptional output per infected cell analysis by transcripts normalized to total DNA. The graphs represent the medians calculated the different and interquartile ranges of different RNAs quantified by ddPCR/ total proviral DNA quantified by IPDA ratio: **A and E)** 5’elongated (LongLTR), **B and F)** unspliced (Pol), **C and G)** polyadenylated (PolyA) and **D and H)** multiply spliced (TatRev). RNA was quantified in resting (**A-D**) and activated (**E-H**) conditions in the HIV+ MS+ (case report, blue circles) and five PWH on ART with undetectable viral load (HIV+ MS-, red circles). Horizontal discontinuous line shows where the ratio is 1.
(TIFF)

S4 Fig Correlation between HIV-1 RNA in culture supernatant and intracellular HIV-1 transcripts. Spearman correlation and linear regression analyses between HIV-1 RNA levels in culture supernatant (quantified by RT-qPCR) and intracellular HIV-1 transcripts (quantified by ddPCR) after 96 hours of activation (with αCD3/CD28 + IL-2). **A.** 5’ elongated (LongLTR), **B.** unspliced (Pol), **C.** polyadenylated (PolyA) and **D.** multiply spliced (TatRev). Spearman correlation coefficient (r) and p-values, as well as linear regression R² values are shown.
(TIFF)

S1 Data Underlying data used for graphing and statistical analyses. This file contains the raw dataset utilized to generate all graphical representations and perform the statistical analyses.
(XLSX)
