# Supplementary material for: Fingolimod increases cellular resistance to HIV-1 infection and limits viral reservoir size in peripheral CD4+ T-cells
Source: PLoS Pathog. 2026 Jun 3;22(6):e1014266. doi: 10.1371/journal.ppat.1014266 (PMC13232849; doi:10.1371/journal.ppat.1014266)
Supplement: S1 Table — (DOCX) [file ppat.1014266.s001.docx]

**S1 Table**. HIV+MS+ leucocytes, lymphocytes, CD8^+^ T-cell and CD4^+^ T-cell historical counts and HIV-1 status
