# Supplementary material for: Fingolimod increases cellular resistance to HIV-1 infection and limits viral reservoir size in peripheral CD4+ T-cells
Source: PLoS Pathog. 2026 Jun 3;22(6):e1014266. doi: 10.1371/journal.ppat.1014266 (PMC13232849; doi:10.1371/journal.ppat.1014266)
Supplement: S2 Table — The values shown are the means at each year. (DOCX) [file ppat.1014266.s002.docx]

**S2 Table**. Annual leucocyte and lymphocyte counts from fingolimod treated participants (HIV+MS+, HIV-MS+). The values shown are the means at each year.
