# Supplementary material for: Fingolimod increases cellular resistance to HIV-1 infection and limits viral reservoir size in peripheral CD4+ T-cells
Source: PLoS Pathog. 2026 Jun 3;22(6):e1014266. doi: 10.1371/journal.ppat.1014266 (PMC13232849; doi:10.1371/journal.ppat.1014266)
Supplement: S3 Table — (DOCX) [file ppat.1014266.s003.docx]

**S3 Table.** List of fluorescent antibodies and markers used for phenotyping cytometry analyses:

| **Antibody** | **Clon** | **Source** | **Identifier** |
| --- | --- | --- | --- |
| CD3 Pacific Blue 450 | SP34-2 | BD Pharmingen | 558124 |
| CD4 BV510 | SK3 | BD Biosciences | 562970 |
| CD14 PerCP Cy5.5 | M5E2 | BD Biosciences | 550787 |
| CD16 BV786 | 3G8 | BD Biosciences | 563689 |
| CD19 PerCP Cy5.5 | HIB19 | BD Biosciences | 561295 |
| CD56 PE | B159 | BD Pharmingen | 555516 |
| CD57 FITC | HNK-1 | BD Biosciences | 333169 |
| NKG2C (CD159c) PE Vio 770 | REA 205 | Miltenyi | 130-120-449 |
| NKG2A (CD159a) APC | REA 110 | Miltenyi | 130-113-563 |
