# Supplementary material for: Fingolimod increases cellular resistance to HIV-1 infection and limits viral reservoir size in peripheral CD4+ T-cells
Source: PLoS Pathog. 2026 Jun 3;22(6):e1014266. doi: 10.1371/journal.ppat.1014266 (PMC13232849; doi:10.1371/journal.ppat.1014266)
Supplement: S4 Table — (DOCX) [file ppat.1014266.s004.docx]

**S4 Table.** List of fluorescent antibodies and markers used for CCR5 cytometry analyses:

| **Antibody** | **Clon** | **Source** | **Identifier** |
| --- | --- | --- | --- |
| Fixable Viability Stain 510 | - | BD Biosciences | 564406 |
| CD3 Alexa Fluor 700 | SK7 | BioLegend | 344822 |
| CD4 Alexa Fluor 647 | MT310 | Santa Cruz Biotechnology | sc-19641 |
| CCR5 PE | 2D7/CCR5 | BD Biosciences | 555993 |
