# Supplementary figures and images for: Fingolimod increases cellular resistance to HIV-1 infection and limits viral reservoir size in peripheral CD4+ T-cells
Source: PLoS Pathog. 2026 Jun 3;22(6):e1014266. doi: 10.1371/journal.ppat.1014266 (PMC13232849; doi:10.1371/journal.ppat.1014266)

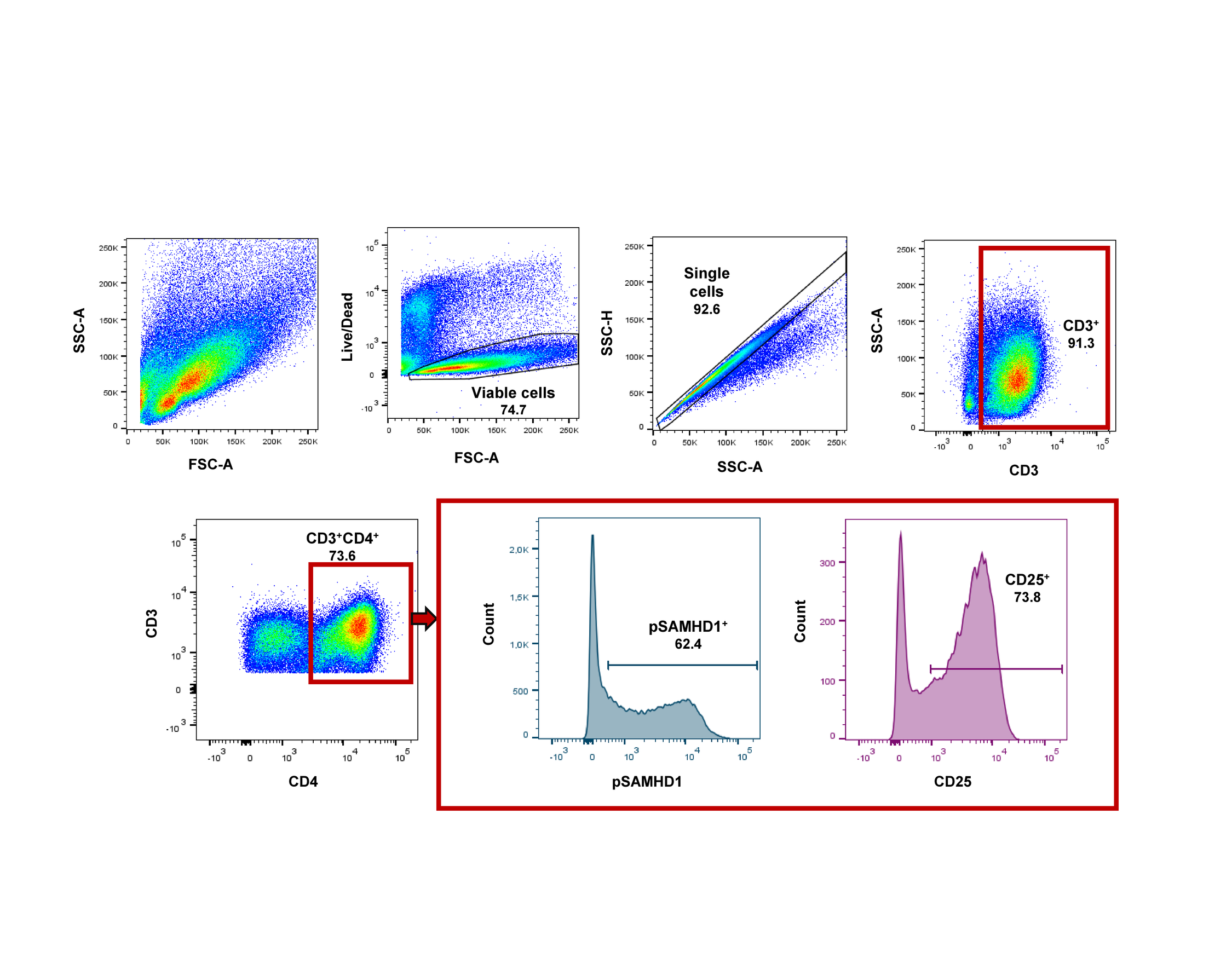

Supplement: S1 Fig — Flow cytometry analysis of PBMCs activated for 72h with PHA + IL-2. It is a representative participant from the HIV- MS- group. (TIFF) [file ppat.1014266.s006.tiff]

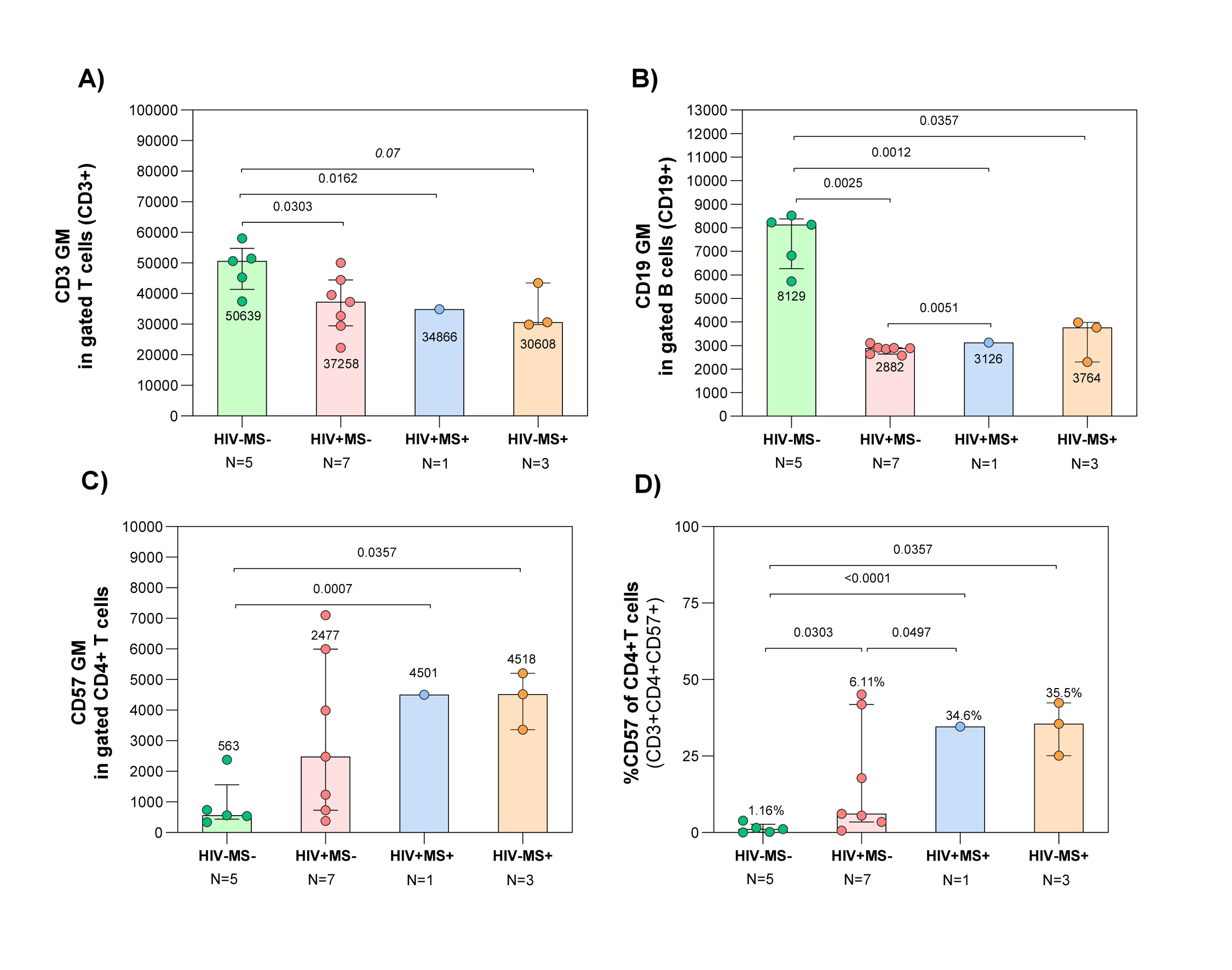

Supplement: S2 Fig — Flow cytometry analysis of PBMCs from non-infected controls (HIV− MS− , green circles), PWH (HIV+ MS − , red circles), case report (HIV+ MS+ , blue circles), and MS patients treated with fingolimod (HIV− MS+ , orange circles). The graph shows median values and interquartile ranges. Geometric mean fluorescence intensity (GM) was used to measure expression levels on membrane. Mann-Whitney test was performed to compare the different groups with N > 1. One sample Wilcoxon test test was performed to compare the HIV+ MS+ values vs HIV+ MS- group. A. CD3 GM in gated CD3+ cells. B. CD19 GM in gated CD19+ cells. C. CD57 GM in gated CD3+CD4+ cells. D. Percentage of CD57+ in gated CD3+CD4+ cells. (TIFF) [file ppat.1014266.s007.tiff]

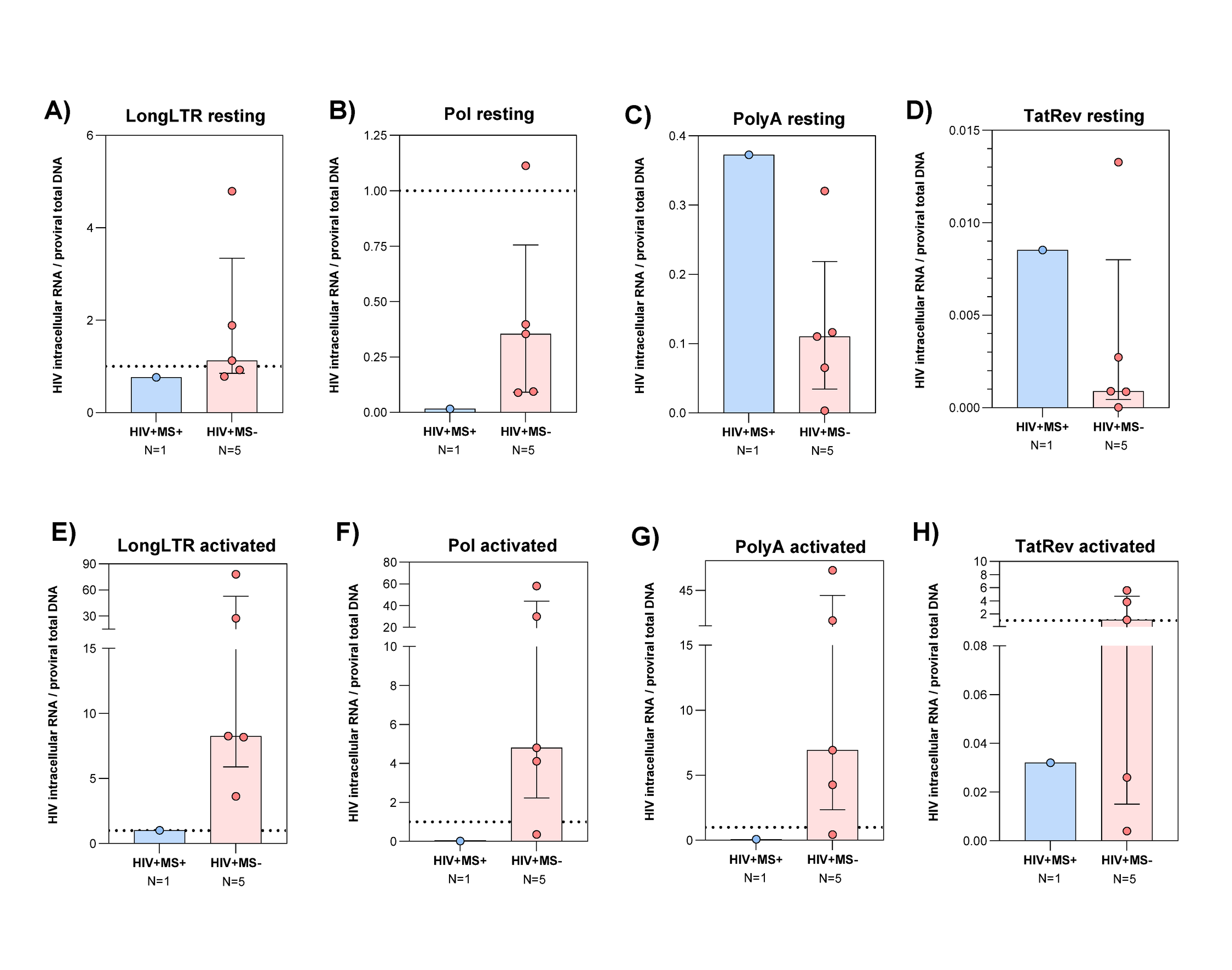

Supplement: S3 Fig — The graphs represent the medians calculated the different and interquartile ranges of different RNAs quantified by ddPCR/ total proviral DNA quantified by IPDA ratio: A and E) 5’elongated (LongLTR), B and F) unspliced (Pol), C and G) polyadenylated (PolyA) and D and H) multiply spliced (TatRev). RNA was quantified in resting (A-D) and activated (E-H) conditions in the HIV+ MS+ (case report, blue circles) and five PWH on ART with undetectable viral load (HIV+ MS-, red circles). Horizontal discontinuous line shows where the ratio is 1. (TIFF) [file ppat.1014266.s008.tiff]

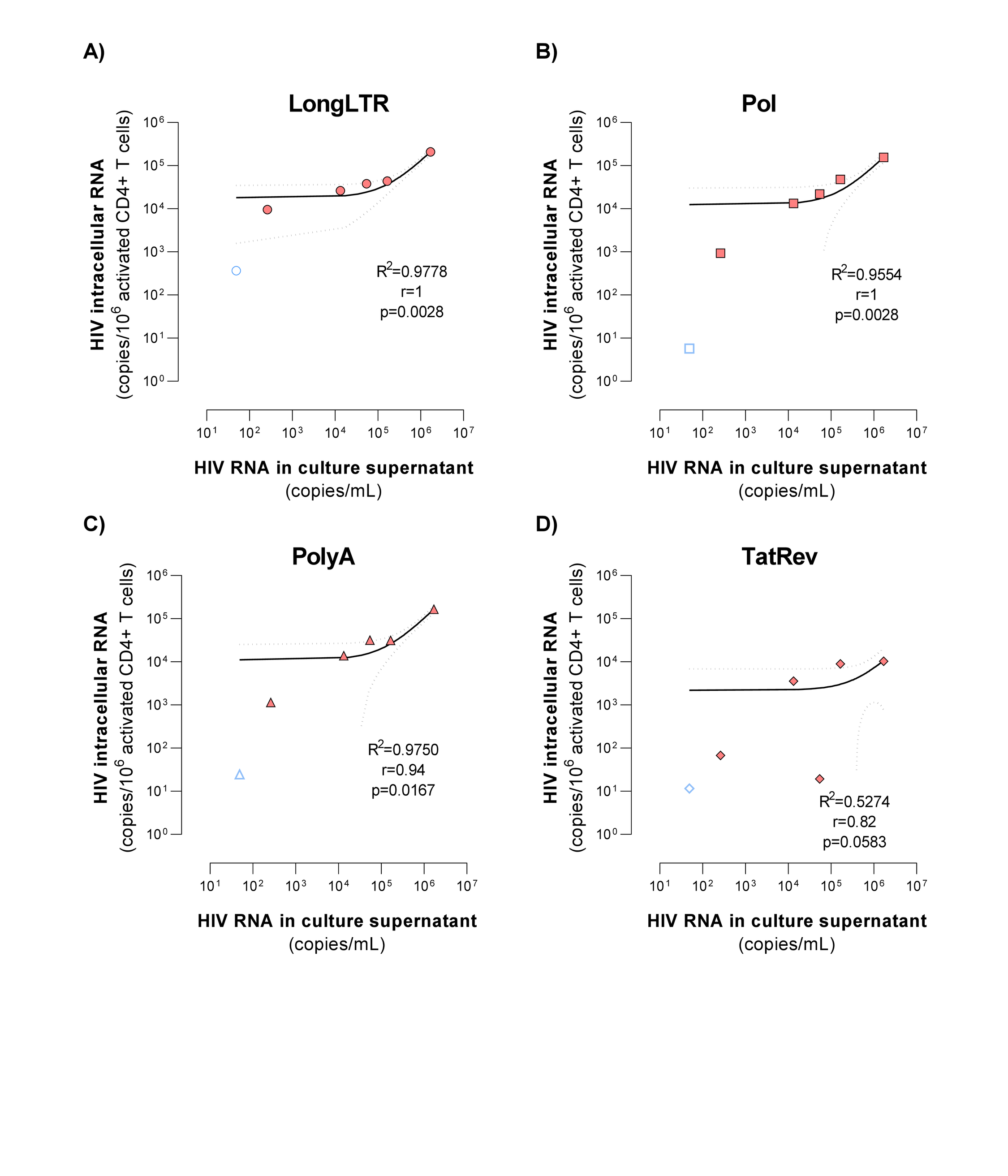

Supplement: S4 Fig — Spearman correlation and linear regression analyses between HIV-1 RNA levels in culture supernatant (quantified by RT-qPCR) and intracellular HIV-1 transcripts (quantified by ddPCR) after 96 hours of activation (with αCD3/CD28 + IL-2). A. 5’ elongated (LongLTR), B. unspliced (Pol), C. polyadenylated (PolyA) and D. multiply spliced (TatRev). Spearman correlation coefficient (r) and p-values, as well as linear regression R² values are shown. (TIFF) [file ppat.1014266.s009.tiff]
